# Global longitudinal strain is a more reproducible measure of left ventricular function than ejection fraction regardless of echocardiographic training

**DOI:** 10.1186/s12947-019-0168-9

**Published:** 2019-09-02

**Authors:** Sigve Karlsen, Thomas Dahlslett, Bjørnar Grenne, Benthe Sjøli, Otto Smiseth, Thor Edvardsen, Harald Brunvand

**Affiliations:** 1 Sørlandet Hospital, Arendal, Norway; 2 St. Olavs Hospital, Trondheim, Norway; 3 Oslo University Hospital, Rikshospitalet, Oslo, Norway

**Keywords:** Global longitudinal strain, Left ventricular ejection fraction, Echocardiographic training

## Abstract

**Background:**

Left ventricular ejection fraction (LVEF) is an established method for evaluation of left ventricular (LV) systolic function. Global longitudinal strain (GLS) by speckle tracking echocardiography seems to be an important additive method for evaluation of LV function with improved reproducibility compared with LVEF. Our aim was to compare reproducibility of GLS and LVEF between an expert and trainee both as echocardiographic examiner and analyst.

**Methods:**

Forty-seven patients with recent Acute Coronary Syndrome (ACS) underwent echocardiographic examination by both an expert echocardiographer and a trainee. Both echocardiographers, blinded for clinical data and each other’s findings, performed image analysis for evaluation of intra- and inter- observer variability. GLS was measured using speckle tracking echocardiography. LVEF was calculated by Simpson’s biplane method.

**Results:**

The trainee measured a GLS of − 19.4% (±3.5%) and expert − 18.7% (±3.2%) with an Intra class correlation coefficient (ICC) of 0.89 (0.74–0.95). LVEF by trainee was 50.3% (±8.2%) and by expert 53.6% (±8.6%), ICC coefficient was 0.63 (0.32–0.80). For GLS the systematic difference was 0.21% (− 4.58–2.64) vs. 4.08% (− 20.78–12.62) for LVEF.

**Conclusion:**

GLS is a more reproducible method for evaluation of LV function than LVEF regardless of echocardiographic training.

## Introduction

Left ventricular ejection fraction (LVEF) is the established method for evaluation of LV systolic function and can be measured by a number of imaging modalities. LVEF by echocardiography has been regarded as a cornerstone in the prediction of outcome and is the most widely available method for evaluation of LV function. It is a vital measurement when determining whether patients benefit from an implantable cardioverter-defibrillator (ICD) or cardiac resynchronization therapy (CRT) [1]. In addition, LVEF is used to define systolic heart failure and has a great impact on the selection of medical treatment [1]. Several echocardiographic methods have been used to measure LVEF but at present, the Simpson’s biplane method is most widely used [2]. Determining LVEF by echocardiography is associated with a high level of inter-observer variability, which to a certain degree can be improved using contrast enhanced echocardiography and 3D echocardiography [3]. Reliability of LVEF depends on image quality and in particular the ability to visualize the endocardial border. Studies have shown that LVEF measured by cardiovascular magnetic resonance imaging (CMR), radionuclide ventriculography and echocardiography is not easily interchangeable [3].

Strain by speckle tracking echocardiography is a technique that utilizes 2-dimensional gray scale images to evaluate both global and regional function of the left ventricle. Peak global longitudinal strain (GLS) may be used to measure systolic function. Previous studies have shown that GLS may both diagnose and exclude acute coronary heart disease better than LVEF [4–6]. In addition, GLS has better intra- and inter-observer reproducibility in post hoc analysis compared to LVEF [4, 7, 8]. Furthermore, GLS may be analyzed in a majority of patients with good feasibility [9] and may be measured as fast as LVEF [4, 10]. Since several studies have shown advantages of GLS compared to LVEF in the evaluation of LV function especially for mild systolic dysfunction [11], GLS is increasingly used in clinical practice. In ESC guidelines for management of acute coronary syndrome in patients presenting without persistent ST-segment elevation, echocardiography is recommended and strain is suggested as a tool to identify reduced regional function [12]. GLS is also recommended used in early detection of cardiotoxicity during chemotherapy [13]. However, it is not well studied how the level of echocardiographic training impact performance of GLS compared to LVEF. It is therefore of interest to study the effect of echocardiographic training on reproducibility of GLS and LVEF.

The aim of this study was to investigate reproducibility of LVEF by Simpson’s biplane and GLS by speckle tracking echocardiography when echocardiographers with different levels of expertise obtain images. Furthermore, we compared inter observer variability of GLS and LVEF between expert and trainee both in image acquisition, image analysis and cross analysis.

## Material and methods

### Study population

We invited 126 surviving patients from a previous study admitted with suspected non-ST elevation acute coronary syndrome (NSTE-ACS) [5, 14] to a five-year follow-up study with echocardiographic examination. Eleven patients declined further participation and 10 did not respond. Patients from this cohort were prospectively included to undergo a double echocardiographic examination by a trainee and an expert examiner, and 47 patients constitute the basis of the present study. The study was approved by the regional ethical committee.

### Echocardiography

To standardize and increase quality of transthoracic echocardiographic examinations (TTE) both the European Association of Echocardiography (EAE) and American College of Cardiology (ACC)/American Heart Association (AHA) have published guidelines regarding echocardiographic and clinical competence [15, 16]. The defined expert in this study was a physician qualified as advanced or level 3-examiner with special interest in speckle tracking echocardiography. This implied a minimum of 12 months of training, 300 performed examinations and 750 interpreted examinations. The trainee was a physician qualified to basic or level 2 which required a minimum of 6 months of training, 150 examinations and 300 interpreted examinations.

Both examiners obtained echocardiographic examinations using a Vivid 7 Scanner (GE Ultrasound, Horten, Norway) with images and cineloops stored digitally. Both sets of echocardiographic examination were performed during the same consultation and the examiners were blinded for each other’s recordings and findings when examining the patients. Three consecutive cycles from three apical and three parasternal image planes were recorded using 2-dimensional gray scale echocardiography. Frame rates were between 55 and 95 frames/second.

Both examiners used the same commercially available software (EchoPAC version 112, GE Ultrasound) for post hoc analysis of echocardiographic recordings on a separate work station. The examiners were blinded for clinical data and echocardiographic findings before analyzing both sets of images. Closure of the aortic valve defined the end of systole and was determined by pulsed Doppler flow in the left ventricular outflow tract (LVOT). Peak systolic strain was defined as the maximum value of peak negative strain (myocardial shortening) or peak positive strain (myocardial lengthening) during systole. GLS by speckle tracking echocardiography was measured manually in a 18-segments LV model as the average segmental value based on three apical imaging planes. LVEF was calculated using Simpson’s biplane method. Image quality for both expert and trainee images was evaluated by the trainee echocardiographer. Image quality was rated poor when < 60% of endocardial border was visible in any standard apical image plane, fair when 60–74% was visualized and good when > 75% of the endocardium was visible. Left ventricular end diastolic diameter was measured perpendicular to the left ventricle in parasternal long axis image measuring the distance from the septal endocardium to the endocardium of posterior wall in the end diastole at the level of the tips of the mitral valve. E wave express early diastolic mitral inflow velocity measured by pulsed doppler. E’ represent early diastolic mitral annular velocity.

### Statistical analysis

Continuous data are presented as mean ± SD or median (inter quartile range). Categorical data are presented as numbers (percentage). All statistical analyses were performed using SPSS 22.0 (SPSS Inc., Chicago, Illinois). Intra class correlation coefficients (ICC) were obtained using two-way mixed model with measures of absolute agreement to describe test reproducibility. ICC of LVEF and GLS were compared using Z-scores. Bland-Altman plots were created to demonstrate inter-observer agreement and were performed by Graphpad Prism ver. 6.05. Linear regression analysis of difference vs mean was used to identify proportional interrater bias. Fixed bias indicates a systemically difference in results between observers, expressed as mean difference analyzed by paired samples T-test.

## Results

### Clinical data

The clinical data of the 47 patients are summarized in Table 1.

**Table 1:** Clinical characteristics

Patient characteristics	*n* = 47
Male	34 (72.3%)
Age	66.2 ± 10.0 (46–89)
Current smoker	9 (19.1%)
Body mass index	26.9 ± 4.1
Systolic blood pressure (mmHg)	141.9 ± 18.2 (100–176)
Diastolic blood pressure (mmHg)	82.7 ± 10.2 (63–104)
Heart rate (beats per minute)	62.5 ± 10.7 (46–91)
NSTE-ACS	24 (51.1%)
Unstable angina pectoris	11 (23.4%)
Non-coronary chest pain	12 (25.5%)
Heart medication and NYHA class at follow up	
ACE-inhibitor	14 (25.9%)
ARB	15 (27.8%)
Calcium antagonist	13 (24.1%)
Beta blocking agent	33 (61.1%)
NYHA I	42 (89.4%)
NYHA II	4 (8.5%)
NYHA III	2 (4.3%)

Categorical data are presented in numbers (%); continuous data as mean ± SD (range); *ACE* = angiotensin converting enzyme, *ARB* = angiotensin receptor blocker, *NSTE-ACS* = Non-ST elevation acute coronary syndrome, *NYHA* = New Yok Heart Association

### Echocardiographic data

GLS and LVEF data in all assessment scenarios are displayed in Table 2. None of the image recordings for LVEF calculation or GLS measurement were rejected due to image quality. Single LV segments were excluded due to suboptimal image quality or poor tracking when performing GLS measurements. More than 95% of all segments were included in the analyses and there was no significant difference in feasibility between the analyses or examiners. Analyzing trainee images, trainee analyzed 17.3 (±1.2) segments versus expert 17.1 (±1.5), *p* = 0.45. In expert images the trainee analyzed 17.4 (±1.0) versus expert 17.6 (±1.1), *p* = 0.38. No images were rejected for LVEF calculation. Results from evaluation of image quality for both echocardiographers are reported in Table 3.

**Table 2:** Echocardiographic parameters presented in all different examiner-analyst scenarios

Scenario		GLS	LVEF	
Expert echocardiographer				
A	Trainee analyst	−19.1% (±3.4)	54.5% (±8.2)	
	Expert analyst	−18.7% (±3.2)	53.6% (±8.6)	
	ICC	0.94 (0.84–0.97)	0.71 (0.47–0.84)	4.04 (*p* < 0.001) †
	Fixed bias	−0.76% (−3.55–2.03)	−0.27% (−16.56–16.02)	0.40 (*p* = 0.692) *
Trainee echocardiographer				
B	Trainee analyst	−19.4% (±3.5)	50.3% (±8.2)	
	Expert analyst	−18.3% (±3.5)	52.1% (±8.9)	
	ICC	0.91 (0.72–0.96)	0.76 (0.57–0.87)	2.49 (p < 0.001) †
	Fixed bias	1.19% (−4.61–2.22)	−1.4% (−15.79–12.98)	2.36 (*p* < 0.02) *
Each operator analyzing their own images				
C	Trainee	−19.4% (±3.5)	50.3% (±8.2)	
	Expert	−18.7% (±3.2)	53.6% (±8.6)	
	ICC	0.89 (0.74–0.95)	0.63 (0.32–0.80)	3.26 (p < 0.001) †
	Fixed bias	−0.97% (−4.58–2.64)	−4.08% (−20.78–12.62)	2.45 (p < 0.02) *
Trainee analyst				
D	Trainee Images	−19.4% (±3.5)	50.3% (±8.2)	
	Expert images	−19.1% (±3.4)	54.5% (±8.2)	
	ICC	0.93 (0.88–0.96)	0.13 (−0.45–0.49)	7.17 (< 0.001) †
	Fixed bias	0.21% (−4.58–2.64)	−4.08% (−20.78–12.62)	− 0.02 (*p* = 0.98) *
Expert analyst				
E	Trainee Images	−18.3% (±3.5)	52.1% (±8.9)	
	Expert images	−18.3% (±3.2)	53.6% (±8.6)	
	ICC	0.91 (0.84–0.95)	0.70 (0.47–0.83)	3.1 (0.002) †
	Fixed bias	0,07 (−3.64–3.78)	−2.17 (−18.16–13.82)	1.84 (*p* = 0.07) *
Trainee imaging versus expert imaging				
F		EDD	E/e’	
	ICC	0.91 (0.85–0.95)	0.92 (0.85–0.95)	
	Fixed bias	−0.43 mm	0.25	

Continuous data are presented as mean ± SD; *GLS* = global longitudinal strain, *LVEF* = left ventricular ejection fraction, *EDD* = left ventricle end diastolic diameter, *E/e’* = early transmitral diastolic inflow divided by early diastolic mitral annular velocity, *ICC* = intra class correlation coefficient (95% confidence interval) compared with Z-score (†). Fixed bias, mean difference (95% confidence interval) compared with paired samples T-test (*). Results in this table are stratified in scenario A-E describing who obtained images and who performed the analysis. Results from comparing GLS and LVEF in each scenario are presented in the Z-score column. Parameters of diastolic function are listed in section F

**Table 3:** Image quality for analysis of LVEF

Echocardiographer	Good	Fair	Poor
Trainee	38 (80.9)	4 (8.5)	5 (10.6)
Expert	38 (80.9)	6 (12.8)	3 (6,4)

Categorical data are presented in numbers (%). *LVEF* = left ventricular ejection fraction. Good = > 75% of visible endocardium. Fair = 60–74% of visible endocardium. Poor = less than 60% of visible endocardium

### Reproducibility and systematic differences

**Fig. 1:**
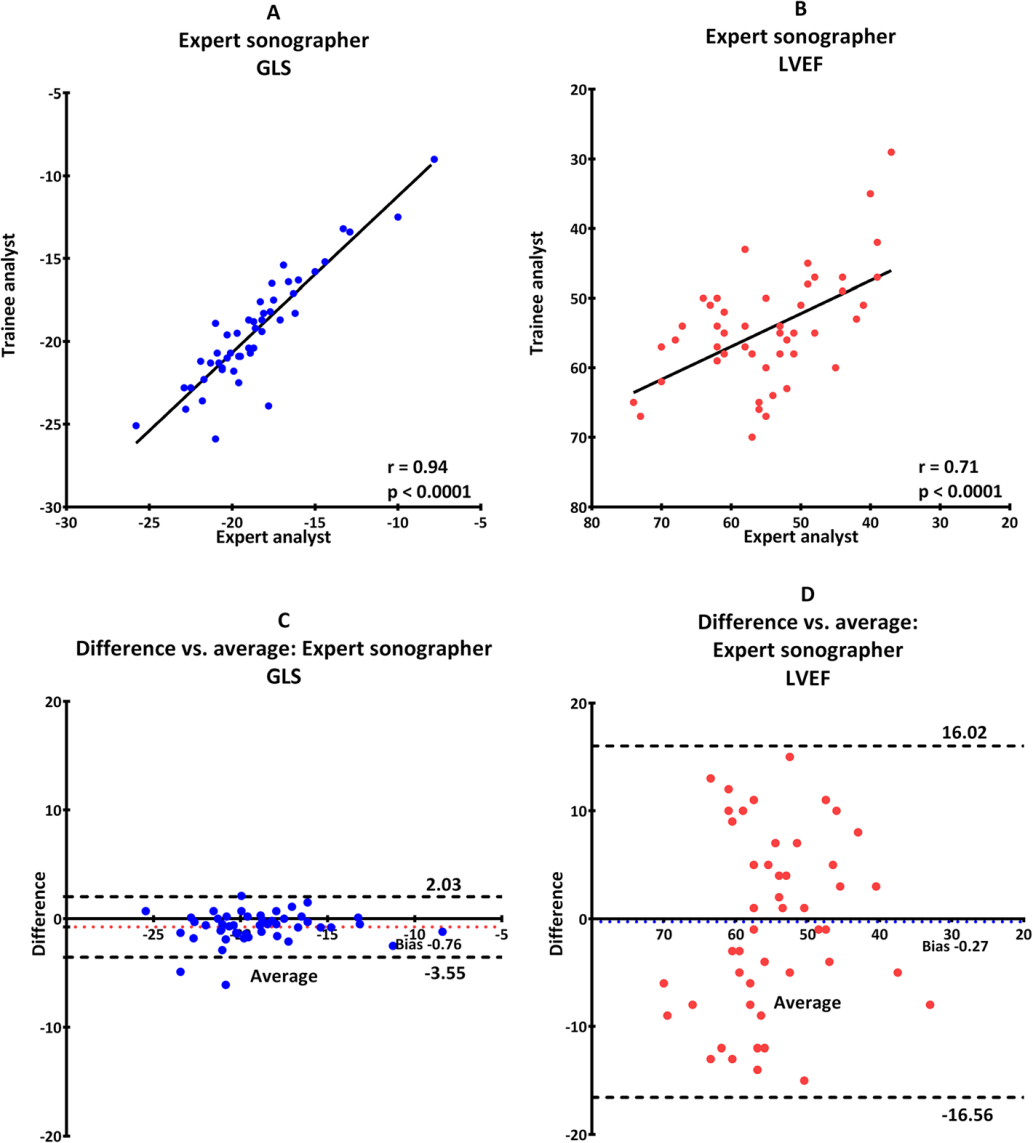
Images obtained by expert echocardiographer analyzed by both trainee and expert. Scatterplot for GLS (**a**) and LVEF (**b**). Bland-Altman plot for GLS (**c**) and LVEF (**d**). Dotted line illustrates 95% confidence interval and colored dotted line illustrate fixed bias

Reproducibility was excellent in general for GLS regardless of level of training in both image acquisition and analysis. It was weakest when trainee and expert compared results after analyzing their own images with an ICC of 0.89 (0.74–0.95) (Table 2, scenario C and Fig. 3). The strongest reproducibility was seen when both analyzed the expert images where ICC was 0.94 (0.84–0.97) (Table 2, scenario A and Fig. 1).

**Fig. 2:**
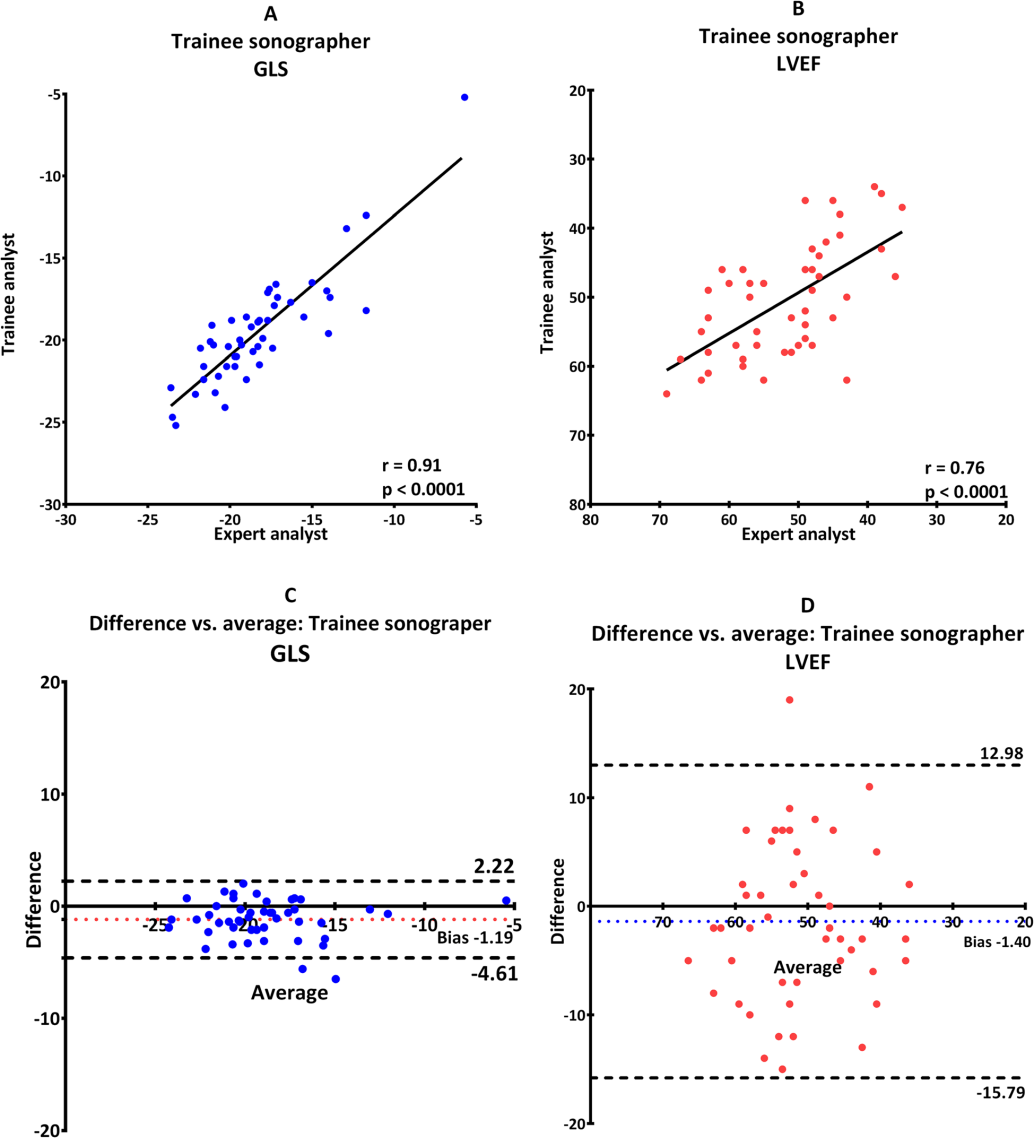
Images obtained by trainee echocardiographer analyzed by both trainee and expert. Scatterplot for GLS (**a**) and LVEF (**b**). Bland-Altman plot for GLS (**c**) and LVEF (**d**). Dotted line illustrates 95% confidence interval and colored dotted line illustrate fixed bias

Reproducibility was good for LVEF and was best when both examiners analyzed trainee images with an ICC of 0.76 (0.57–0.87) (Table 2, scenario B and Fig. 2). When the trainee analyzed both set of images the ICC was only 0.13 (− 0.45–0.49) (Table 2, scenario D and Fig. 4).

There was a significant difference in reproducibility between GLS and LVEF in favor of GLS as listed in Table 2 in all echocardiographer-analyst scenarios.

**Fig. 3:**
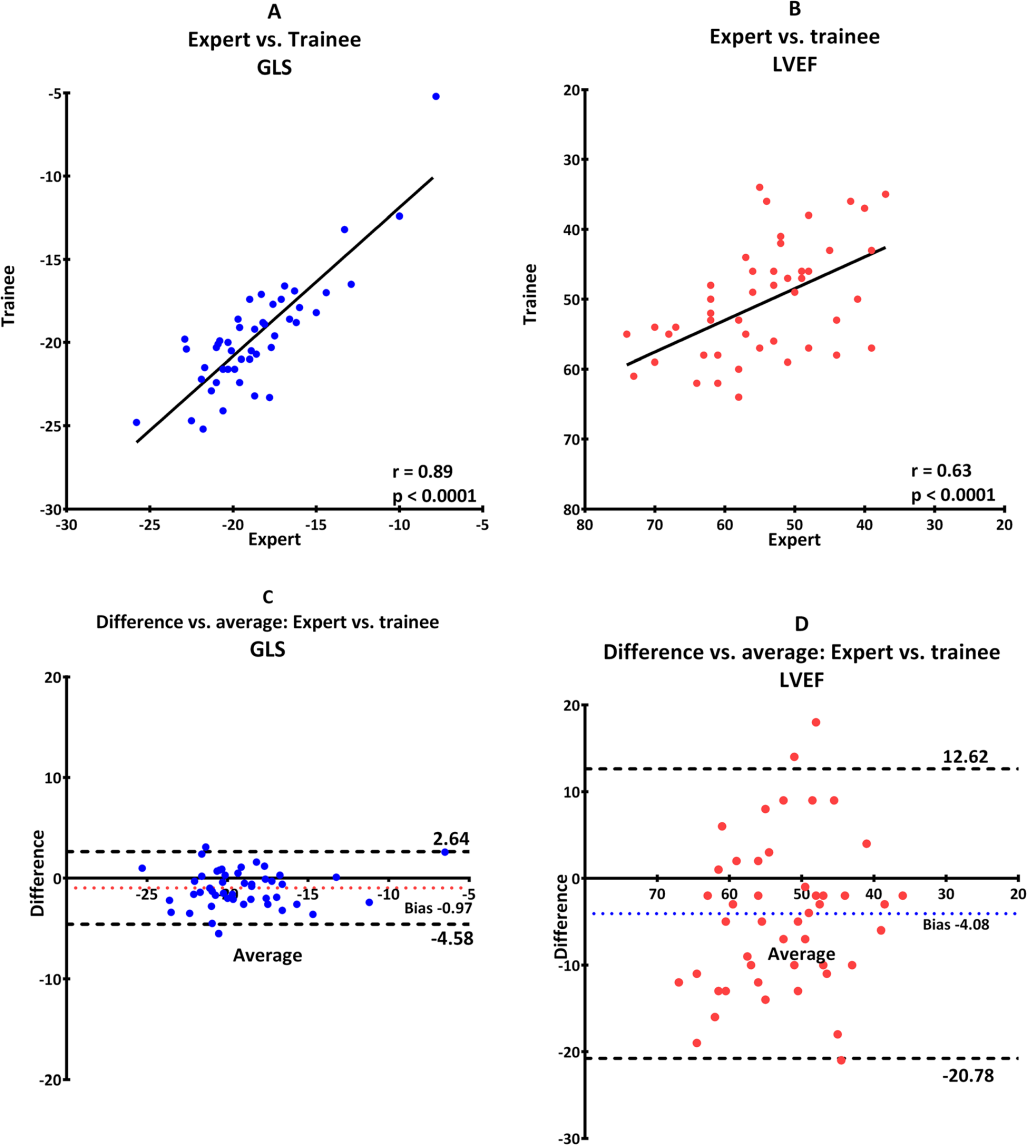
Analysis of expert analysis of expert images versus trainee analysis of trainee images. Scatterplot for GLS (**a**) and LVEF (**b**). Bland-Altman plot for GLS (**c**) and LVEF (D). Dotted line illustrates 95% confidence interval and colored dotted line illustrate fixed bias

**Fig. 4:**
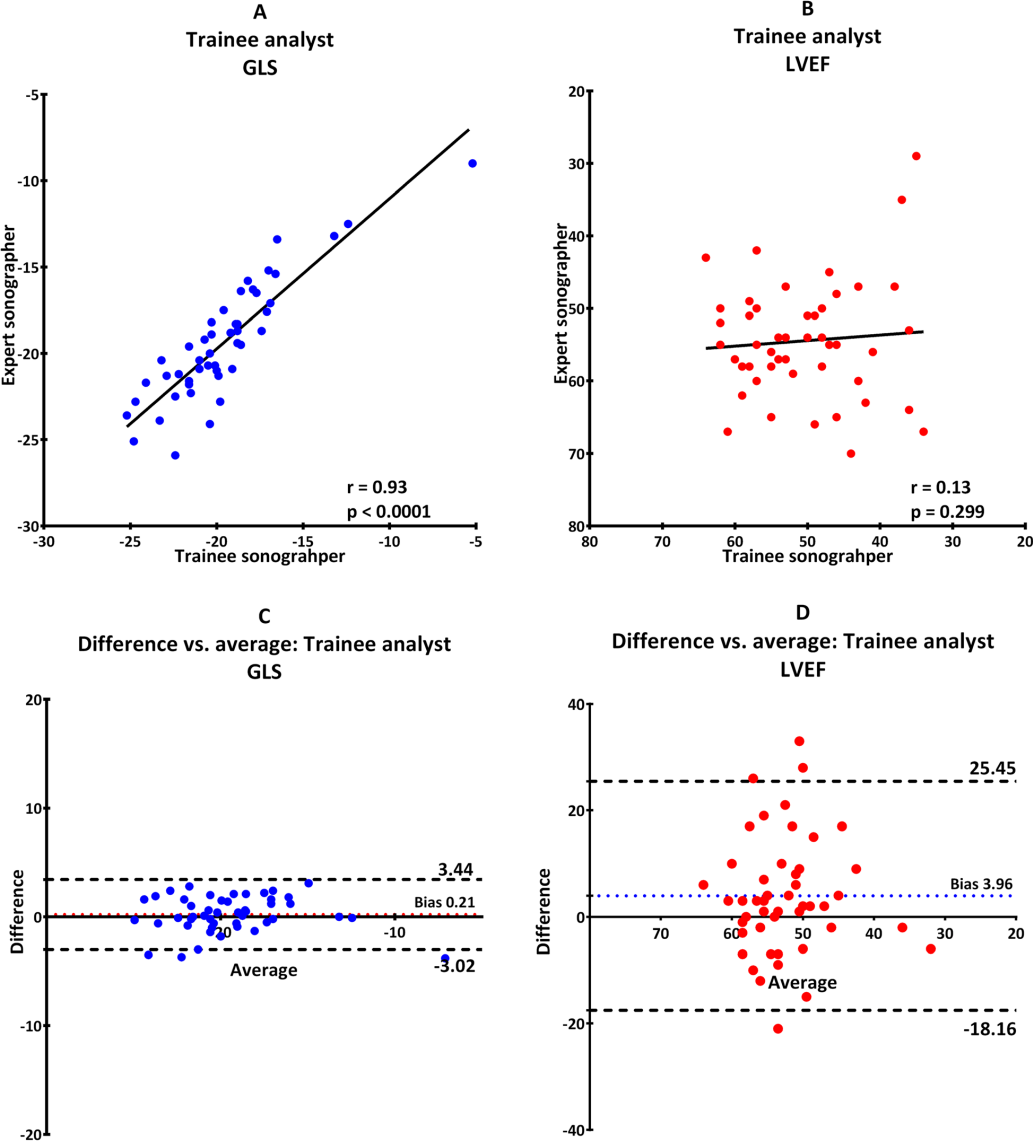
Trainee analyzing images obtained by both expert and trainee. Scatterplot for GLS (**a**) and LVEF (**b**). Bland-Altman plot for GLS (**c**) and LVEF (**d**). Dotted line illustrates 95% confidence interval and colored dotted line illustrate fixed bias

**Fig. 5:**
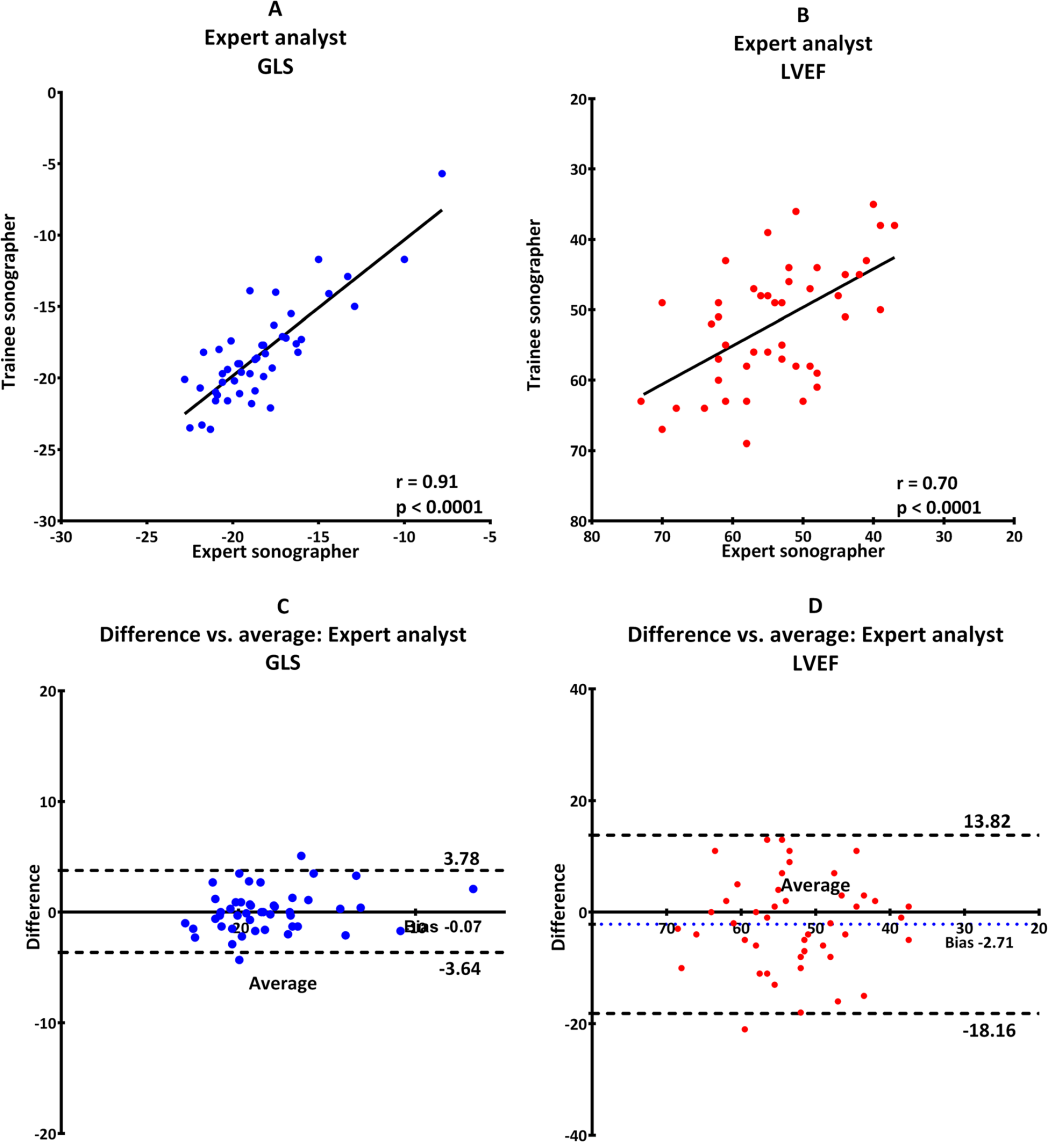
Expert analyzing images obtained by both expert and trainee. Scatterplot for GLS (**a**) and LVEF (**b**). Bland-Altman plot for GLS (**c**) and LVEF (**d**). Dotted line illustrates 95% confidence interval and colored dotted line illustrate fixed bias

Furthermore, there was no proportional bias for GLS or LVEF, which indicate that the measurements agreed equally through the entire range of measurements. Fixed bias was present in all scenarios indicating a systematic difference in results of both GLS and LVEF (Fig. 1, 2, 3, 4, 5). There was a significant difference in fixed bias between GLS and LVEF when both examiners analyzed trainee images (Table 2, scenario B and Fig. 2) and when both examiners analyzed their own images (Table 2, scenario C and Fig. 3). In both scenarios, the fixed bias was higher in LVEF analysis.

**Fig. 6:**
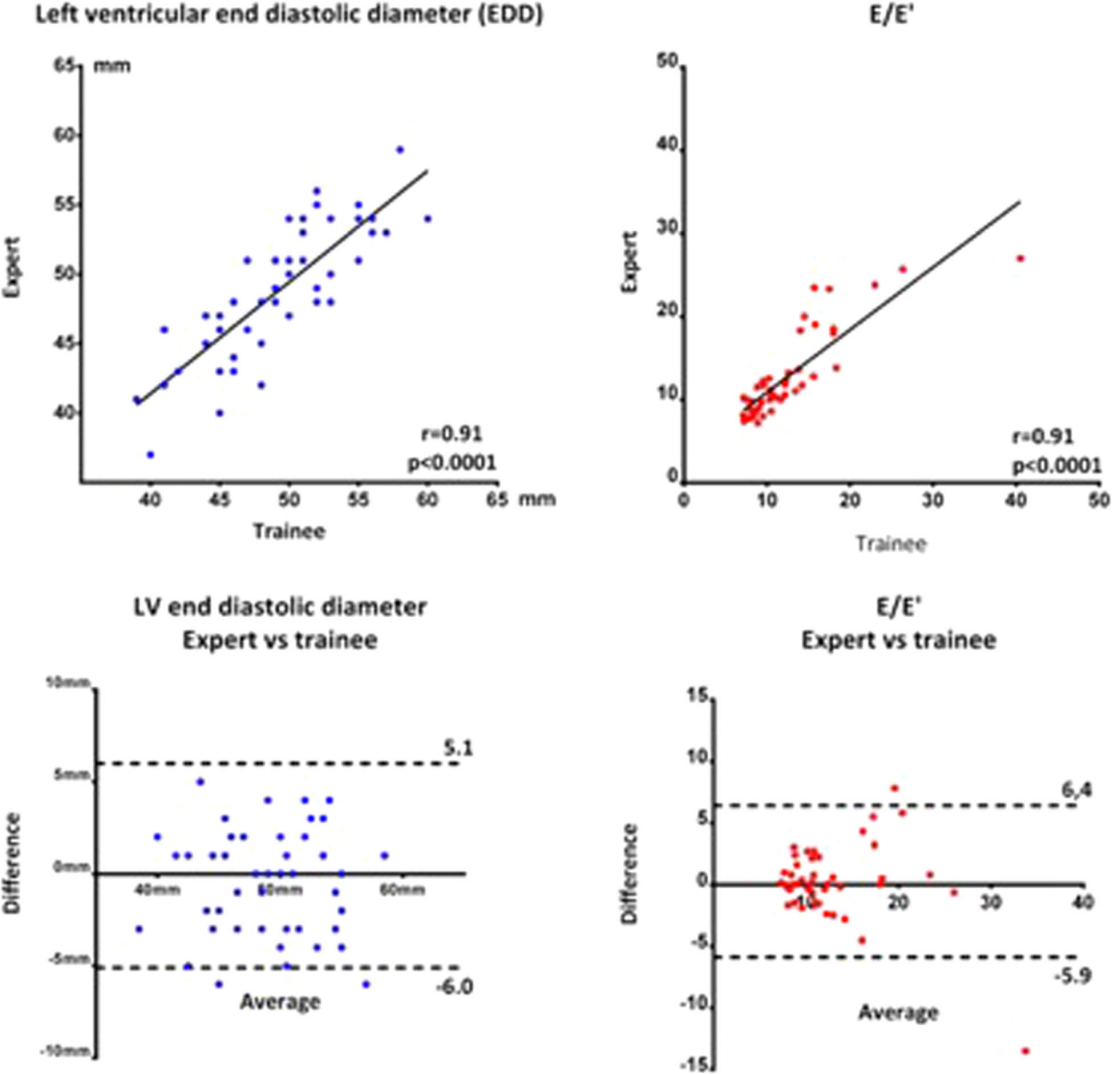
Correlation of EDD and E/E’ in trainee analysis of trainee and expert images displayed in scatterplot and Bland-Altman plot. Dotted line illustrates 95% confidence interval

When trainee comparing left ventricular end diastolic diameter in expert and trainee images ICC was 0.91 (0.85–0.95) with a systematic bias of − 0.43 mm. ICC of E/e’ was 0.92 (0.85–0.95) with a systematic bias of 0.25 (Fig. 6).

## Discussion

To our knowledge, this is one of very few studies to demonstrate that GLS is a more reproducible method for evaluation of LV systolic function than LVEF regardless of echocardiographic training. These findings support the emerging clinical use of GLS as an additional and incremental diagnostic tool in specific myocardial diseases.

A number of previous studies have compared the ability of GLS and LVEF to detect small reductions in LV function, particularly in ischemic heart disease [10, 14, 17–19]. In these studies, GLS and segmental strain had better ability than LVEF to predict infarct size and segmental viability in patients with myocardial infarction [18, 20], diagnose coronary artery occlusion in patients with NSTEMI [5, 21], exclude coronary artery disease in patients with chest pain [6], predict risk of ventricular arrhythmias [22] and predict mortality [4, 11]. The use of GLS in stress echocardiography increases diagnostic precision compared to LVEF and wall motion scoring, even for novice readers [23]. Earlier studies have described inter- and intra-observer variability in LVEF [24] and GLS outperforming LVEF [4, 7]. In these studies, image readers were regarded as experts. However, despite several advantages of GLS compared to LVEF in clinical practice, LVEF is still the most used method for evaluation of LV systolic function. In order to use GLS in clinical practice it is important to know to how echocardiographic training may affect the analysis of GLS. We found that measurement of GLS by echocardiography in clinical practice is a highly reproducible method independent of echocardiographic training and significantly better reproducible than LVEF. There may be several reasons for this finding.

When measuring GLS and calculating LVEF there are numerous sources of error in both image acquisition and image analysis that may affect measurements and results. The sources of error include how each operator records and analyzes images offline. Level of echocardiographic training influence both image acquisition and image analysis and may potentially lead to high variability for both LVEF and GLS.

Obtaining images suitable for both LVEF and GLS analysis requires several technical considerations [2]. In LVEF calculation, we need high-quality visualization of the endocardial border in both apical 4-chamber and 2-chamber views. The images should display LV cavity with minimum foreshortening. Timing of end systole and diastole is critical. End-diastole is defined as the first frame after mitral valve closure or the frame which LV dimension is the largest. [25] End-systole is defined as the frame after aortic valve closure or the frame in which the cardiac dimension or volume is smallest. [25] Error in these steps will lead to miscalculation of cavity volume and LVEF. As illustrated in Table 3, it seems that the expert echocardiographer generally was able to achieve better images for LVEF analysis. The differences in image quality and visibility of endocardial border may depend on factors as gain setting, focus depth and sector width. Small differences in cavity foreshortening and apical transducer rotation might introduce variability in volume calculation as well. Variability may increase with increasing LVEF due to larger variation in endocardial border between end diastole and end systole.

A major limitation of LVEF in patients with myocardial infarction is that the Simpson’s biplane method is based on an assumption of symmetric LV geometry. The presence of regional myocardial dysfunction as a result of myocardial infarction alters LV geometry. [2] As a consequence, the Simpson’s biplane method by echocardiography may partly fail to measure LVEF with precision, and level of echocardiographic experience may affect how LVEF is measured. Correlation between GLS and LVEF is reported higher in healthy subjects than in patients with myocardial infarction and heart failure. [26] Our study population is a heterogeneous group regarding ischemic myocardial pathology (Table 1) and degree of LV dysfunction (Table 4).

**Table 4:** Distribution of patients according to LVEF

LVEF	35–40%	41–54%	> 55%
Trainee echocardiographer	11 (23.4)	18 (38.3)	18 (38.3)
Expert echocardiographer	8 (17.0)	15 (31.9)	24 (51.1)

Categorical data are presented in numbers (%). LVEF = left ventricular ejection fraction

GLS does not rely on geometric assumptions but measures myocardial function with precision as we have demonstrated previously [5, 10, 14, 17, 21]. Strain by speckle tracking measure directly segmental myocardial deformation of the LV in a 16-segment model. Average deformation of LV is expressed as GLS. LVEF describes LV systolic function indirectly on the basis of changes in calculated LV volume during the systole. In addition, GLS may be more sensitive than LVEF to changes in long-axis shortening, which makes GLS useful in evaluation of LV function where LVEF is preserved [27, 28]. After the region of interest (ROI) is set in strain measurement, speckle tracking is performed automatically by the respective software.

Image acquisition for strain analysis by speckle tracking has different sources of error [29]. Recognition and elimination of acoustic phenomena as reverberation and acoustic shadowing is important. Tracking of these phenomena will result in underestimation of true deformation [29, 30]. Since strain by speckle tracking is essentially angle independent, this can to a certain degree be eliminated by adjusting probe position. The software performs automated speckle tracking frame by frame which imply that the frame rate needs to be optimized. A frame rate between 40 and 80 frames per second (FPS) is often recommended [29, 30]. Low frame rate and tachycardia may result in undersampling where systolic events are missed, resulting in underestimation of true deformation [30].

Images suitable for strain analysis may be easier to obtain than images for endocardial tracing. Manual tracing of the endocardium in two image planes may be performed with significant variability between observers due to differences in defining the endocardial border in both end diastole and end systole even in high quality images. Variability is influenced by differences in image acquisition. Since GLS on the other hand is a direct and objective measurement of myocardial deformation and function, this may reduce variability between echocardiographers.

Our findings demonstrate that GLS is a more reproducible parameter regardless of echocardiographic training and image quality compared to LVEF. Level of training is probably more important for LVEF calculation. Our results are similar to a study of Medvedkovsky et al. who addressed the same issue but with another vendor and software [31] and of Negishi et al. [8]. The findings in this study supports the use of GLS in clinical practice as an important supplement in describing LV function with low variability between observers even among echocardiographic trainees.

## Limitations

At present, there is no industrial standard for strain analysis among different echocardiographic machine vendors. GLS may at the present time therefore vary between vendors and measured results may not be interchangeable. Identical equipment should be used comparing examinations. In the future, it is likely that there will be an industry standard for strain regardless of vendor and GLS may then be measured and compared with any echocardiographic machine [32]. At present there are limited software available that allows automated LVEF measurement that could reduce inter observer reproducibility.

## Conclusions

The present study demonstrates that GLS is a more reproducible method for evaluation of LV function than LVEF regardless of echocardiographic training.

## Data Availability

The datasets during and/or analyzed during the current study is available from the corresponding author on reasonable request.

## Abbreviations

ACC: American College of Cardiology
ACE: Angiotensin converting enzyme
ACS: Acute coronary syndrome
AHA: American Heart Association
ARB: Angiotensin receptor blocker
CMR: Cardiovascular magnetic resonance imaging
CRT: Cardiac resynchronization therapy
EAE: European Association of Echocardiography
EDD: End diastolic diameter
ESC: European society of cardiology
FPS: Frames per seconds
GLS: Global longitudinal strain
ICC: Intra class correlation coefficient
ICD: Implantable cardioverter-defibrillator
LV: Left ventricle
LVEF: Left ventricular ejection fraction
LVOT: Left ventricular outflow tract
NSTE-ACS: Non-ST elevation acute coronary syndrome
NYHA: New Yok Heart Association
ROI: Region of interest
SD: Standard deviation
TTE: Transthoracic echocardiographic examination
